# Dexmedetomidine reduces acute kidney injury in high-risk but not low-risk patients after non-cardiac surgery: secondary analysis of a randomized controlled trial

**DOI:** 10.1007/s00540-026-03661-8

**Published:** 2026-01-17

**Authors:** Xin-Quan Liang, Mei-Lin Li, Chun-Jing Li

**Affiliations:** Department of Anesthesiology, Peking University First Hospital, No. 8 Xishiku St., Xicheng District, Beijing, 100034 China

**Keywords:** Acute kidney injury, Dexmedetomidine, Non-cardiac surgery, High-risk patients

## Abstract

**Purpose:**

Acute kidney injury (AKI) is a significant postoperative complication associated with poor long-term outcomes. Dexmedetomidine, a selective α₂-adrenergic agonist with anti-inflammatory properties, may protect the kidney during non-cardiac surgery. This study examined whether intraoperative dexmedetomidine reduces AKI in both high-risk and low-risk patients.

**Methods:**

This was a secondary analysis of a randomized double-blind placebo-controlled trial. Patients aged ≥ 60 years scheduled for elective non-cardiac surgery expected to last ≥ 2 h under general anesthesia were enrolled and classified as low-risk or high-risk using the General Surgery Acute Kidney Injury Risk Index. Participants were randomly allocated to receive intraoperative dexmedetomidine (loading dose 0.6 μg/kg over 10 min before induction, followed by 0.5 μg/kg/h until 1 h before surgery end) or normal saline. The primary endpoint was AKI incidence within 7 postoperative days.

**Results:**

Among high-risk patients, AKI occurred in 12.6% (13/103) of the dexmedetomidine group versus 23.4% (25/107) of controls (RR 0.54, 95% CI 0.29 to 1.00, *P* = 0.043); after multivariable adjustment, dexmedetomidine remained independently associated with lower AKI risk (OR 0.44, 95% CI 0.20 to 0.98, *P* = 0.045). In contrast, low-risk patients showed no significant difference with or without dexmedetomidine (7.9% vs 9.6%; RR 0.82, 95% CI 0.44 to 1.55, *P* = 0.543; adjusted OR 0.65, 95% CI 0.30 to 1.38, *P* = 0.260). Urological surgery was an independent predictor of AKI across the entire cohort.

**Conclusion:**

Intraoperative dexmedetomidine was associated with lower risk of AKI in high-risk but not in low-risk patients undergoing non-cardiac surgery.

**Supplementary Information:**

The online version contains supplementary material available at https://doi.org/10.1007/s00540-026-03661-8.

## Introduction

Acute kidney injury (AKI) is a major complication after surgery, with reported incidences ranging from 6 to 27% [1]. Older patients are prone to AKI because of age-related decline in kidney function. In these population, the incidence of AKI may reach 14.9% [2]. AKI is associated with increased morbidity, mortality, and long-term adverse events such as chronic kidney disease [3, 4].

Surgery-induced inflammation and oxidative responses are considered the underlying mechanism of AKI [5, 6]. Dexmedetomidine, a highly selective alpha-2 agonist, is known for its sedative, anxiolytic, analgesic, and sympatholytic properties. In animal studies, dexmedetomidine has been shown to protect renal function by inhibiting inflammation, reducing oxidative stress, promoting autophagy, and inhibiting apoptosis [7–10]. Recent meta-analyses showed that perioperative dexmedetomidine can reduce the incidence of AKI after cardiac surgery [11, 12], and it also confers renoprotection in septic patients with systemic inflammatory response syndrome [13]. Collectively, these evidences suggest that dexmedetomidine may preserve renal function in patients at high-risk of AKI.

The effect of dexmedetomidine on renal function after non-cardiac surgery patients remains inconsistent. Two retrospective studies reported that dexmedetomidine was associated with a lower incidence of postoperative AKI in elderly patients undergoing major joint arthroplasty and in hypertensive patients undergoing non-cardiac surgery [14, 15]. However, another retrospective study found that dexmedetomidine did not reduce AKI incidence after thoracic surgery [16]. A meta-analysis also showed that intraoperative dexmedetomidine infusion was not associated with AKI in non-cardiac surgical patients [17], but this conclusion is limited by the small sample sizes of the included trials and by heterogeneity among populations with both low and high AKI risk.

Compared with cardiac or septic patients, those undergoing non-cardiac surgery experience less severe surgery-induced stress and carry a lower baseline risk of AKI. Based on these evidences, we hypothesized that dexmedetomidine confers renoprotection primarily in patients at high risk for AKI. The present study was therefore designed to determine whether intraoperative dexmedetomidine exerts different renoprotective effects in low-risk versus high-risk patients after non-cardiac surgery.

## Materials and methods

The present study was a secondary analysis of a randomized double-blind placebo-controlled trial. The study protocol was approved by the Clinical Research Ethics Committee of Peking University First Hospital (No. 2015-987) and registered with the Chinese Clinical Trial Registry on 1 December 2015 (www.chictr.org.cn; registration number ChiCTR-IPR-15007654). Written informed consent, which included permission for secondary data analysis, was obtained from every patient or the patient’s legal representative in the original trial.

### Participants

Inclusion criteria were: (1) patients aged 60 years or above; (2) scheduled to undergo elective non-cardiac surgery with an expected duration 2 h or more under general anesthesia.

Exclusion criteria were: (1) refusal or inability to provide written informed consent; (2) previous history of schizophrenia, epilepsy or Parkinson’s disease; (3) visual, hearing, language or other barrier that precluded communication and delirium assessment; (4) history of traumatic brain injury or neurosurgery; (5) severe bradycardia (heart rate less than 40 beats per minute), sick sinus syndrome, or atrioventricular block of degree 2 or above without pacemaker; (6) severe hepatic dysfunction (Child–Pugh grade C); (7) renal failure (requirement for renal replacement therapy).

Present study also excluded patients without record of preoperative or postoperative serum creatinine values.

### Randomized, blinding and study drug administration

An independent biostatistician generated a 1:1 randomization sequence using SAS 9.2 software package (SAS Institute, Cary, NC, USA). This sequence was concealed in sequentially numbered, opaque envelopes and kept by a study coordinator who had no role in patient recruitment, data collection, perioperative care or postoperative follow-up.

During the study, trial drugs were prepared by the coordinator according to randomization results. An attending anesthesiologist was designated for administration of study drugs.

The trial drugs—either 200 μg dexmedetomidine or an equivalent volume of normal saline—were diluted to 50 mL with normal saline (final concentration 4 μg/mL for dexmedetomidine). All solutions were colorless and supplied in identical syringes.

Patients, investigators, and healthcare team members remained blinded to treatment assignment throughout the study. To protect patient safety, allocation could be unmasked if a severe adverse event or unexpected clinical deterioration occurred; any such unmasking was recorded in the case-report form.

### Intervention, anesthesia and perioperative care

In the dexmedetomidine group, a loading dose of the trial drug (0.15 ml/kg, equivalent to 0.6 μg/kg dexmedetomidine) was administered over 10 min before anesthesia induction, followed by a continuous infusion of 0.125 ml/kg/hr (0.5 μg/kg/hr dexmedetomidine) until 1 h before the end of surgery. Patients in the control group received volume-matched normal saline at the same rates and durations.

Attending anesthesiologists were permitted to reduce or discontinue the trial-drug infusion if any of the following occurred: (1) severe bradycardia or hypotension that persisted despite routine treatment; (2) new-onset atrioventricular block that failed to improve after routine treatment; or (3) any other condition deemed clinically necessary by the anesthesiologist. All changes to the infusion rate and the reason for each change were documented.

Anesthesia was induced with intravenous propofol and sufentanil, and maintained with intravenous propofol and sufentanil, as well as inhalation of a 1:1 nitrous oxide: oxygen mixture. Rocuronium and/or cisatracurium provided muscle relaxation. Depth of anesthesia was monitored by the Bispectral index (BIS), which was maintained between 40 and 60. Blood-pressure variations was maintained within ± 20% of baseline. Nasopharyngeal temperature was maintained between 36.0 ∘C and 37.0∘C. Fluid infusion and blood transfusion were performed according to routine practice.

All patients were transferred to the post-anesthesia care unit (PACU) or the ICU before returning to a general ward. Postoperative analgesia was provided with patient-controlled intravenous analgesia (PCIA) consisting of morphine 0.5 mg/mL, programmed to deliver a background infusion of 0.5 mL/h and a 2 mL bolus with an 8-min lockout interval. Intravenous non-steroidal anti-inflammatory drugs and/or oral tramadol could be administered as needed. All other perioperative management was performed according to routine clinical practice.

### Primary outcome

The primary outcome was the incidence of AKI within the first 7 postoperative days. AKI was diagnosed and staged according to the Kidney Disease Improving Global Outcomes (KDIGO) criteria based on changes in serum creatinine: stage 1, an increase in serum creatinine to 1.5–1.9 times the baseline levels within the previous 7 days or by ≥ 0.3 mg/dl (≥ 26.5 µmol/l) within 48 h; stage 2, an increase in serum creatinine to 2.0–2.9 times the baseline levels within the previous 7 days; and stage 3, an increase in serum creatinine increases to ≥ 3.0 times the baseline levels or to ≥ 4 mg/dl (353.6 µmol/L) within the previous 7 days or initiation of renal replacement therapy or in patients < 18 years a decrease in eGFR < 35 ml/min/1.73 m^2^ [18].

### Secondary outcomes

Secondary outcomes included the time to first onset of postoperative AKI, the severity of AKI identified by AKI stage, the incidence of non-AKI complications within 30 days and the length of hospital stay after surgery. Non-AKI complications were defined as any new-onset medical condition that adversely affected patient recovery and required therapeutic intervention within 30 days of surgery, that is, grade II or greater in the Clavien–Dindo classification [19].

### Classification of patients with low-risk and high-risk AKI

An American study reported nine independent factors to assess the AKI Risk in non-cardiac surgical patients, including age above 56 years old, male, active congestive heart failure, ascites, hypertension, emergency surgery, intraperitoneal surgery, renal insufficiency-mild or moderate (preoperative serum creatinine value > 1.2 mg/dl), and diabetes mellitus-oral or insulin therapy [20]. General Surgery Acute Kidney Injury Risk Index classes are established based on the number of risk-factors the patient possesses. The incidence of AKI is 0.2% in class I (zero, one, or two risk factors), 0.8% in class II (three risk factors), 2.0% in class III (four risk factors), 3.6% in class IV (five risk factors), and 9.5% in class V (six or more risk factors). This preoperative risk prediction model demonstrated an excellent predictive capability, with a c statistic of 0.80. In our study, we pre-specified Class III (≥ 4 risk factors) or higher as “high-risk” because this threshold was selected based on the original validation study of this index, which showed a clinically significant jump in AKI incidence at the Class III level.

### Data collection

Baseline data included demographics, diagnosis, comorbidities, smoking, alcoholism, preoperative medication, New York Heart Association (NYHA) classification, American Society of Anesthesiologists (ASA) classification, and AKI risk index class. Baseline renal function was evaluated with preoperative serum creatine and estimate Glomerular Filtration Rate (eGFR).

Intraoperative data included type and duration of anesthesia, use of anesthetics and analgesics, use of glucocorticoids, type and duration of surgery, estimated blood loss, fluid infusion and blood transfusion, as well as adverse events.

Postoperative data included consumption of morphine in patient-controlled analgesia, supplemental sedatives and analgesics, length of postoperative in-hospital stay, occurrence of non-AKI complications within 30 days, and all-cause 30-day mortality. Investigators who performed patient recruitment and data collection were trained before the study period.

### Statistical analysis

#### Sample size calculation

Sample size calculation in previous study was based on the hypothesis that intraoperative dexmedetomidine could decrease the incidence of postoperative delirium (310 patients in dexmedetomidine group and 310 patients in control group) [21]. As a secondary analysis, we planned to investigate the effect of dexmedetomidine on postoperative AKI in different risk patients. Thus, we excluded patients without record of preoperative or postoperative serum creatinine values. We finally enrolled 401 patients in AKI low-risk class (203 patients in dexmedetomidine group and 198 patients in control group), and 210 patients in AKI high-risk class (103 patients in dexmedetomidine group and 107 patients in control group).

#### Statistical analysis

For baseline balance, normality of continuous data was tested by Kolmogorov–Smirnov method in prior. Data with normal distribution were presented as mean ± standard deviations (SD) and differences between groups were compared by independent t sample. Data without normal distribution were presented as median (IQR) and differences between groups were compared by Mann–Whitney U test. Categorical data was presented by number (percentage) and differences between groups were compared by Chi-square test, continuity correction chi-square tests, or Fisher exact tests.

For primary outcome, the incidence of AKI within postoperative 7 days was compared with a chi-square test. Estimated effect size was reported in the form of relative risk (RR) with 95% confidence interval (CI) in intention-to-treat analysis. For secondary outcomes, incidence of non-AKI complication was analyzed using the chi-square tests, continuity correction chi-square tests, or Fisher exact tests, with differences between groups expressed as relative risk and 95% CI. Time-to-event results were analyzed with Kaplan–Meier survival analyses, with differences between groups tested using log-rank tests; univariable Cox proportional hazards models were used to calculated hazard ratios and 95%CIs. Discrete variables were analyzed with the Mann–Whitney U test; median difference (and 95% CI) was calculated with the Hodges–Lehmann estimator.

Factors that might be associated with the development of postoperative AKI were screened with univariate logistic regression analyses. Confounders were selected based on the following criteria: (1) baseline and perioperative variables with statistical significance (i.e., *P* < 0.05) in univariable logistic regression analysis, which were conducted to identify potential associations with AKI, (2) imbalanced variables between patients with/without dexmedetomidine, and (3) clinically important factors. These confounders were then entered simultaneously into a single multivariable logistic-regression model for each risk class (high-risk and low-risk) to estimate the risk-adjusted effect of intra-operative dexmedetomidine on post-operative AKI. SPSS 23.0 software package (SPSS Inc, Chicago, Ill) was used for statistical analysis. Two-sided P values of less than 0.05 were considered statistically significant.

## Results

### Participants

**Fig. 1:**
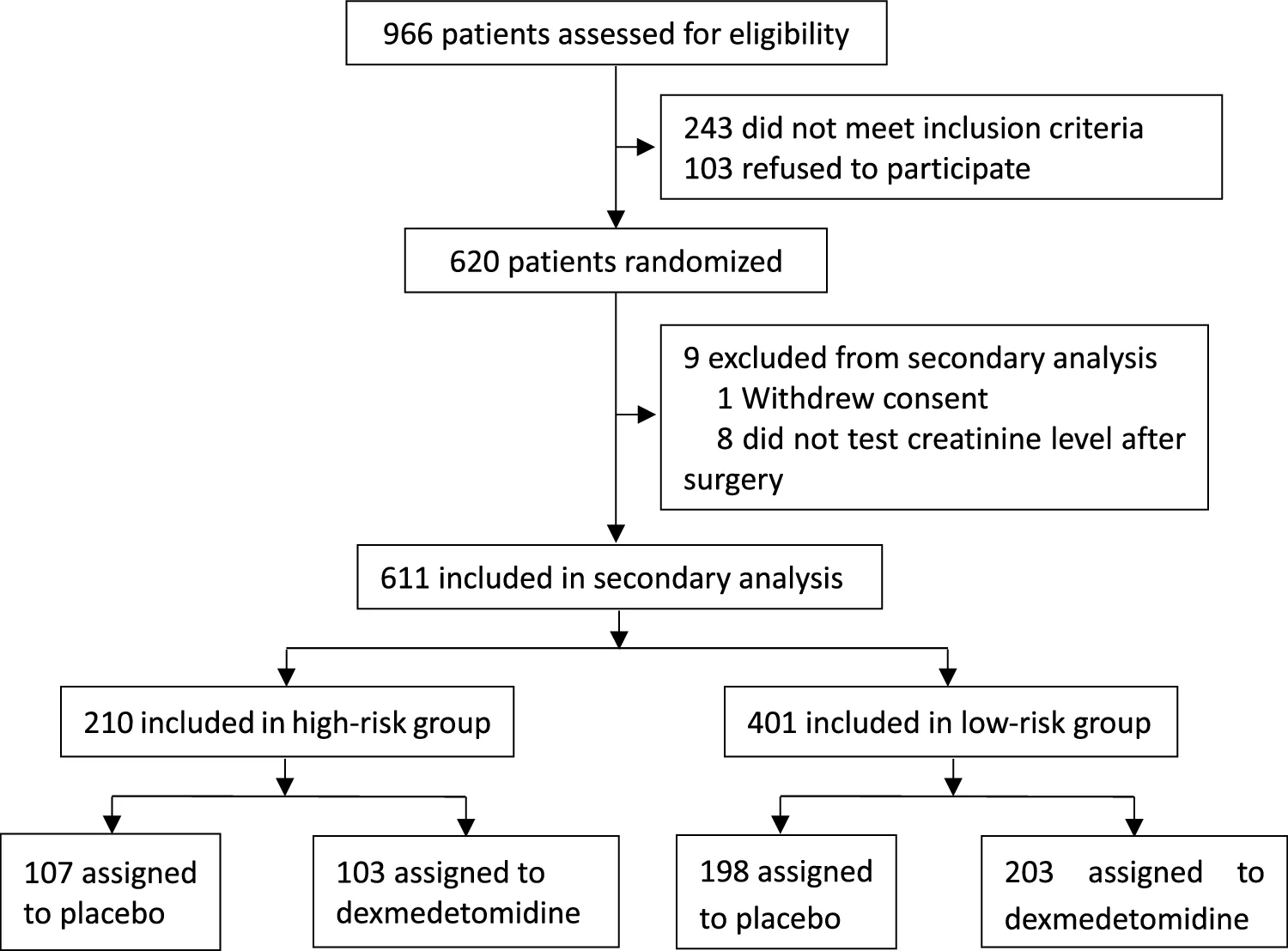
Trial diagram

A total of 619 patients were screened. 8 patients were excluded because of no record of serum creatinine, Fig. 1. According to General Surgery Acute Kidney Injury Risk Index, 210 patients were allocated to high-risk class and 401 patients to low-risk class. High-risk patients exhibited an older age profile, a higher proportion of male patients, greater comorbidity burden, higher baseline creatinine, and more frequent use of ACEI/ARB and aspirin. Surgically, they underwent more abdominal (notably urological) procedures but received fewer vasopressors and NSAIDs, with reduced transfusion needs and less urine output (Table S1 and S2).

For high-risk class, 103 patients received dexmedetomidine and 107 patients were treated with placebo. Mean age and baseline serum creatine were similar between two groups. Other demographic and baseline variables showed no statistical significance between two groups. For low-risk class, 203 patients received dexmedetomidine and 198 patients were treated with placebo. Demographic and baseline variables were also well-balanced between the two groups (Table 1).

**Table 1:** Demographic and baseline data

	Low-risk patients (*n* = 401)			High-risk patients (*n* = 210)		
	Dexmedetomidine (*n* = 203)	Placebo (*n* = 198)	*P* value	Dexmedetomidine (*n* = 103)	Placebo (*n* = 107)	*P* value
Age, years	67 (63, 74)	67 (64, 73)	0.642	69 (64, 76)	69 (64, 75)	0.746
Female sex	108 (53.2)	99 (50.0)	0.521	17 (16.5)	19 (17.8)	0.810
Body mass index, kg m^−2^	23.88 (21.56, 26.12)	23.61 (21.30, 26.24)	0.915	24.80 (22.86, 26.64)	24.80 (22.23, 27.04)	0.828
Preoperative comorbidity						
Hypertension	63 (31.0)	60 (30.3)	0.874	83 (80.6)	80 (74.8)	0.312
Coronary heart disease	24 (12.4)	19 (9.9)	0.471	18 (17.5)	27 (25.2)	0.171
Previous stroke	17 (8.4)	15 (7.6)	0.768	10 (9.7)	18 (16.8)	0.130
Arrhythmia	12 (5.9)	19 (9.6)	0.167	12 (11.7)	11 (10.3)	0.751
COPD	1 (0.5)	2 (1.0)	0.983	3 (2.9)	3 (2.8)	> 0.999
Diabetes mellitus	18 (8.9)	15 (7.6)	0.638	52 (50.5)	46 (43.0)	0.276
Renal dysfunction^a^	2 (1.0)	2 (1.0)	> 0.999	6 (5.8)	5 (4.7)	0.708
Chronic smoking^b^	46 (22.7)	48 (24.2)	0.708	31 (30.1)	29 (27.1)	0.631
Alcoholism^c^	22 (10.8)	24 (12.1)	0.687	19 (18.4)	18 (16.8)	0.757
Preoperative medication						
ACEI/ARB	21 (10.3)	27 (13.6)	0.310	23 (22.3)	26 (24.3)	0.736
Aspirin	12 (5.9)	7 (3.5)	0.263	8 (7.8)	12 (11.2)	0.395
NYHA classification			0.722			0.722
I	148 (72.9)	148 (72.9)		63 (61.2)	68 (63.6)	
II	55 (27.1)	48 (24.2)		40 (38.8)	39 (36.4)	
III	0 (0)	2 (1.0)		0 (0)	0 (0)	
ASA classification			0.549			0.132
I	34 (16.7)	36 (18.2)		2 (1.9)	5 (4.7)	
II	158 (77.8)	154 (77.8)		85 (82.5)	73 (68.2)	
III	11 (5.4)	8 (4.0)		16 (15.5)	29 (27.1)	
CCI^d^	4 (3, 5)	4 (4, 5)	0.158	5 (4, 5)	5 (4, 5)	0.910
Serum creatine (ummol/L)	82.0 (73.0, 92.0)	83.0 (74.0, 91.0)	0.931	95.0 (87.0, 105.4)	96.0 (83.0, 109.0)	0.982
eGFR (ml/min/1.73m^2^)	63.52 (51.86, 76.56)	62.32 (52.10, 73.87)	0.748	54.46 (42.29, 65.80)	52.82 (44.01, 66.90)	0.947
Preoperative assessment, point						
AKI risk class^e^, class	2 (1, 2)	2 (1, 2)	0.364	3 (3, 4)	3 (3, 4)	0.866

Data are median (interquartile range), *n* (%), or mean ± SD. Numbers in square brackets indicate patients with missing data. *P* values in bold indicate < 0.05

COPD chronic obstructive pulmonary disease, ACEI angiotensin-converting enzyme inhibitor, ARB angiotensin receptor blocker, NYHA New York heart association, ASA American society of anesthesiologists, CCI charlson comorbidity index, eGFR estimated glomerular filtration rate

^a^Glomerular filtration rate lower than 30 ml/min/1.73m^2^

^b^Smoking half a pack (10 cigarettes)/day for at least 1 year, either former or current smoker

^c^Two drinks or more daily or weekly consumption of the equivalent of 150 ml of alcohol

^d^Including age-adjusted

^e^Based on general surgery acute kidney injury risk index

In low-risk class, a higher proportion of patients in the control group underwent mini-invasive surgery compared to those in the dexmedetomidine group (*P* = 0.029, Table 2). Sufentanil and propofol consumption were lower in low-risk patients receiving dexmedetomidine than in those receiving placebo (*P* = 0.007 and *P* < 0.001, respectively, Table 2). The mean BIS was lower in patients who received dexmedetomidine in both low-risk (*P* < 0.001) and high-risk classes (*P* < 0.001). These patients also exhibited higher urine output in both low-risk and high-risk categories (*P* = 0.003 and *P* = 0.043, respectively, Table 2). Other intraoperative and postoperative variables were comparable between the two groups.

**Table 2:** Intraoperative and postoperative data

	Low-risk patients (*n* = 401)			High-risk patients (*n* = 210)		
	Dexmedetomidine (*n* = 203)	Placebo (*n* = 198)	*P* value	Dexmedetomidine (*n* = 103)	Placebo (*n* = 107)	*P* value
Intraoperative data						
Duration of anesthesia, min	277 (205, 350)	295 (218, 366)	0.101	277 (215, 339)	258 (184, 369)	0.551
Duration of surgery, min	198 (132, 274)	210 (147, 285)	0.209	193 (145, 260)	180 (123, 300)	0.640
Site of surgery			0.171			0.925
Intrathoracic	49 (24.1)	47 (23.7)		5 (4.9)	6 (5.6)	
Intraabdominal	108 (53.2)	120 (60.6)		95 (92.2)	99 (92.5)	
Spinal and extremital	46 (22.7)	31 (15.7)		3 (2.9)	2 (1.9)	
Urological surgery^a^	46 (22.7)	33 (16.7)	0.131	43 (41.7)	48 (44.9)	0.649
Type of surgery			**0.029**			0.313
Open	66 (32.5)	45 (22.7)		9 (8.7)	14 (13.1)	
Mini-invasive^b^	137 (67.5)	153 (77.3)		94 (91.3)	93 (86.9)	
Medications during anesthesia						
Dosage of study drug, μg	114 (88, 144)	120 (95, 152)	0.098	127 (103, 158)	112 (87, 151)	0.098
Dose of propofol, mg	760 (574, 1070)	970 (700, 1322)	**< 0.001**	830 (670, 1130)	950 (663, 1400)	0.136
Dose of sufentanil, μg	71 (54, 95)	80 (63, 102)	**0.007**	72 (58, 89)	74 (55, 114)	0.395
Use of vasopressors^c^	72 (35.5)	75 (37.9)	0.616	33 (32.0)	27 (25.2)	0.275
Use of vasodilators^d^	23 (11.3)	27 (13.6)	0.485	12 (11.7)	15 (14.0)	0.608
Use of NSAIDs^e^	7 (3.4)	7 (3.5)	0.962	4 (3.9)	3 (2.8)	0.959
Use of 5-HT3 receptor antagonists^f^	177 (87.2)	173 (87.4)	0.956	94 (91.3)	89 (83.2)	0.080
Use of low-dose glucocorticoids^g^	197 (97.0)	190 (96.0)	0.554	100 (97.1)	104 (97.2)	> 0.999
Total infusion, ml	2300 (1600, 3350)	2200 (1600, 3100)	0.186	2600 (1850, 3350)	2300 (1600, 3600)	0.424
Use of colloids, ml^h^	0 (0, 500)	0 (0, 500)	0.732	500 (0, 500)	0 (0, 500)	0.243
Blood transfusion	39 (19.2)	36 (18.2)	0.791	13 (12.6)	12 (11.2)	0.753
Estimated blood loss, ml	100 (50, 300)	100 (50, 300)	0.682	100 (50, 275)	100 (50, 300)	0.817
Urine output, ml	600 (300, 900)	450 (300, 700)	**0.003**	475 (250, 808)	350 (200, 600)	**0.043**
Mean BIS value (%)^i^	52 (49, 56)	55 (51, 59)	**< 0.001**	51 (48, 54)	55 (50, 59)	**< 0.001**
Adverse events						
Hypotension	81 (39.9)	84 (42.4)	0.608	41 (39.8)	32 (29.9)	0.132
Hypertension	26 (12.8)	32 (16.2)	0.340	11 (10.7)	17 (15.9)	0.267
Arrhythmia	2 (1.0)	5 (2.5)	0.426	2 (1.9)	3 (2.8)	> 0.999
Postoperative data						
PCIA morphine, mg	49 (39, 50)	48 (40, 50)	0.825	50 (42, 50)	50 (41, 50)	0.297
Supplemental analgesics within 5 days						
Use of NSAIDs^j^	98 (48.3)	102 (51.5)	0.517	29 (28.2)	34 (31.8)	0.567
Use of oral tramadol	5 (2.5)	3 (1.5)	0.748	1 (1.0)	3 (2.8)	0.641
Other sedatives within 5 days						
Use of midazolam	6 (3.0)	7 (3.5)	0.743	1 (1.0)	5 (4.7)	0.232
Use of dexmedetomidine	4 (2.0)	3 (1.5)	> 0.999	3 (2.9)	6 (5.6)	0.533
Use of propofol	1 (0.5)	0 (0)	> 0.999	0 (0)	0 (0)	> 0.999

Data are median (interquartile range), *n* (%) or mean ± SD. Numbers in square brackets indicate patients with missing data. *P* value in bold indicates < 0.05

*NSAIDs* non-steroid anti-inflammatory drugs, *5-HT3* 5-hydroxytryptamine type 3, *BIS* bispectral index, *PCIA* patient-controlled intravenous analgesia

^a^Included radical nephrectomy, partial nephrectomy, radical nephroureterectomy, radical cystectomy, ureteral reimplantation, radical prostatectomy

^b^Included laparoscopic and thoracoscopic surgery

^c^Included ephedrine, phenylephrine, metaraminol, norepinephrine, epinephrine and dopamine

^d^Included nicardipine and urapidil

^e^Included flurbiprofen axetil (50–100 mg) and parecoxib (40 mg)

^f^Included tropisetron (5 mg) and ondansetron (4 mg)

^g^Dexamethasone (5–10 mg) or methylprednisolone (40 mg) for the prevention of postoperative nausea and vomiting

^h^Included hydroxyethyl starch and/or succinylated gelatin

^i^Monitored by Bispectral Index (BIS) with data collected at 1-min intervals from end of anaesthesia induction to end of surgery

^j^Included parecoxib (40 mg) or flurbiprofen axetil (50 mg), administered within 5 days of surgery

### Primary outcome

**Fig. 2:**
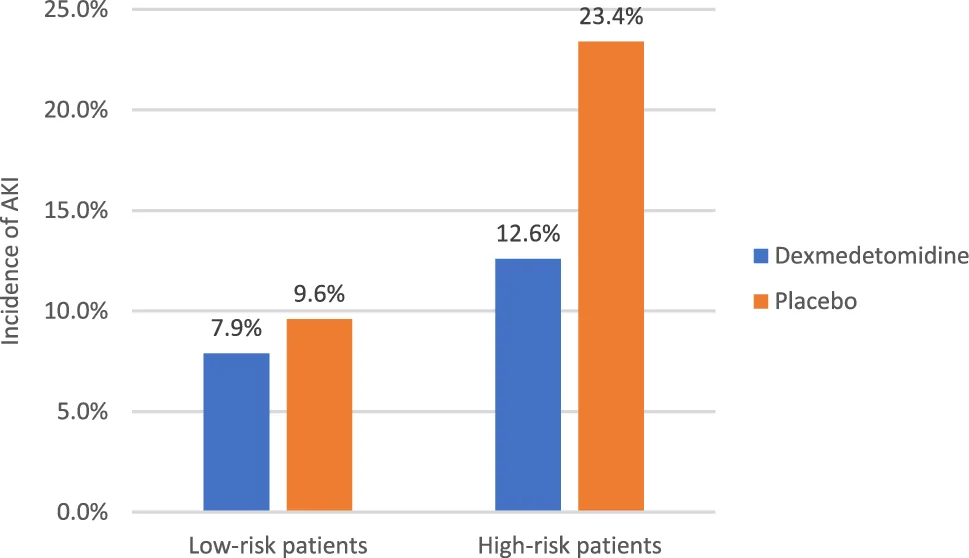
The incidence of AKI in low-risk and high-risk groups. AKI, acute kidney injury

For high-risk patients, the incidence of AKI was 12.6% (13/103) in dexmedetomidine group which was statistical lower than 23.4% (25/107) in control group (RR 0.54, 95% CI 0.29 to 1.00, *P* = 0.043, Fig. 2, Table 3).

**Table 3:** Effectiveness outcomes

	Low-risk patients (*n* = 401)				High-risk patients (*n* = 210)			
	Dexmedetomidine (*n* = 203)	Placebo (*n* = 198)	Median difference, RR, or HR (95% CI)^a^	*P* value	Dexmedetomidine (*n* = 103)	Placebo (*n* = 107)	Median difference, RR, or HR (95% CI)^a^	*P* value
Primary outcome								
Incidence of AKI within 7 days^a^	16 (7.9)	19 (9.6)	0.82 (0.44,1.55)	0.543	13 (12.6)	25 (23.4)	0.54 (0.29, 1.00)	**0.043**
Secondary outcomes								
Onset time to AKI, days	1 (1,1)	1 (1,1)	1.18 (0.59,2.35)	0.641	1 (1,1)	1 (1,1)	1.19 (0.60, 2.36)	0.619
AKI stage^b^				0.841				**0.024**
I	14 (6.9)	17 (8.6)			9 (8.7)	23 (21.5)		
II	2 (1.0)	2 (1.0)			3 (2.9)	1 (0.9)		
III	0 (0)	0 (0)			1 (1.0)	1 (0.9)		
Incidence of non-AKI complication^c^	30 (14.8)	36 (18.2)	1.23 (0.79,1.92)	0.358	15 (14.6)	24 (22.4)	0.65 (0.36, 1.17)	0.143
Length of stay in hospital after surgery, day	8 (6,11)	8 (6,11)	1.12 (0.92,1.36)	0.287	8 (5,12)	8 (6,11)	0.97 (0.74, 1.28)	0.834

Data are median (interquartile range), *n* (%), or mean ± SD. Numbers in square brackets indicate patients with missing data

*RR* relative risk, *HR* hazard ratio, *CI* confidence interval, *AKI* acute kidney injury

^a^Diagnosed according to the KIDGO creatinine criteria, that is the presence and any of the following condition: (1) serum creatinine increase ≥ 0.3 mg/dl (≥ 26.5 µmol/l) within 48 h; or (2) increase of serum creatinine ≥ 1.5 times of basal value within 7 days of onset

^b^stage 1: serum creatinine increases 1.5–1.9 times baseline or ≥ 26.5 µmol/L; stage 2: serum creatinine increases 2.0–2.9 times baseline; stage 3: serum creatinine increases 3.0 times baseline or ≥ 353.6 µmol/L or initiation of renal replacement therapy

^c^Defined as new-onset medical condition with unfavorable impact on patient recovery and requiring therapeutic intervention within 30 days of surgery; individual non-AKI complications are listed in Table S3 (supporting information)

For low-risk patients, the incidence of AKI in dexmedetomidine group had no statistical difference in comparison with control group (7.9% [16/203] vs. 9.6% [19/198], RR = 0.82, 95% CI 0.44 to 1.55, *P* = 0.543, Fig.2, Table 3).

### Secondary outcomes

In high-risk patients, dexmedetomidine mainly reduced the incidence of stage I AKI (stage I: 8.7% vs. 21.5%; stage II: 2.9% vs. 0.9%; stage III 1.0% vs. 0.9%; *P* = 0.024, Table 3). Median onset time of AKI, incidence of non-AKI complications and length of stay in hospital after surgery were comparable between patients with dexmedetomidine or not both in low-risk and high-risk patients (All *P* values > 0.05, Table 3).

### Sensitivity analysis

For high-risk patients, univariate analysis screened out four potential risk factors of AKI. After adjustment of these confounders, dexmedetomidine was associated with a decreased risk of AKI (adjusted OR = 0.44, 95% CI 0.20 to 0.98, *P* = 0.045, Table 4). Urological surgery (OR = 6.21, 95% CI 2.49 to 15.50, *P* < 0.001) and higher Charlson Comorbidity Index (OR = 1.98, 95% CI 1.35 to 2.89, *P *< 0.001) were independent risk factors of AKI (Table 4).

**Table 4:** Risk factors of acute kidney injury in high-risk patients

Variables	Univariate analysis		Multivariable analysis	
	OR (95% CI)	*P* value	OR (95% CI)	*P* value
Intraoperative dexmedetomidine (yes)	0.47 (0.23, 0.99)	0.046	0.44 (0.20, 0.98)	0.045
CCI^a^ (per point increase)	1.58 (1.17, 2.14)	0.003	1.98 (1.35, 2.89)	< 0.001
Mini-invasive surgery^b^ (yes)	0.18 (0.02, 1.41)	0.103	0.16 (0.01, 2.02)	0.156
Urological surgery^c^ (yes)	5.72 (2.54, 12.85)	< 0.001	6.21 (2.49, 15.50)	< 0.001

*OR* odds ratio, *CI* confidence interval, *CCI* charlson comorbidity index

^a^Including age-adjusted

^b^Included laparoscopic and thoracoscopic surgery

^c^Included radical nephrectomy, partial nephrectomy, radical nephroureterectomy, radical cystectomy, ureteral reimplantation, radical prostatectomy

For low-risk patients, univariate analysis screened out four potential risk factors of AKI. Both in univariate and multivariable analysis, dexmedetomidine was not associated with AKI (adjusted OR = 0.65, 95% CI 0.30 to 1.38, *P* = 0.260, Table 5). Urological surgery was independent risk factors of AKI (OR = 9.03, 95% CI 4.06 to 20.10, *P* < 0.001, Table 5).

**Table 5:** Risk factors of acute kidney injury in low-risk patients

Variables	Univariate analysis		Multivariable analysis	
	OR (95% CI)	*P* value	OR (95% CI)	*P* value
Intraoperative use of dexmedetomidine (yes)	0.81 (0.40,1.62)	0.544	0.65 (0.30, 1.38)	0.260
CCI^a^ (per point increase)	1.26 (0.97, 1.63)	0.087	1.11 (0.79, 1.56)	0.563
Mini-invasive surgery^b^ (yes)	0.14 (0.03,0.61)	0.008	0.38 (0.08, 1.83)	0.230
Urological surgery^a^ (yes)	10.61 (4.99, 22.55)	< 0.001	9.03 (4.06, 20.10)	< 0.001

*OR* odds ratio, *CI* confidence interval, *CCI* charlson comorbidity index

^a^Including age-adjusted

^b^Included laparoscopic and thoracoscopic surgery

^c^Included radical nephrectomy, partial nephrectomy, radical nephroureterectomy, radical cystectomy, ureteral reimplantation, radical prostatectomy

## Discussion

This study found that, in the elderly undergoing non-cardiac major surgery, intraoperative dexmedetomidine reduced the rate of postoperative AKI in high-risk patients but not low-risk patients.

In this study, we stratified patients using the General Surgery Acute Kidney Injury (AKI) Risk Index, a well-validated predictor derived from a large U.S. national dataset (c-statistic 0.80) that relies on routinely available clinical variables [20]. The 7-day AKI incidence showed a step-wise increase, from 8.2% in Class I–II patients to 17.6% in Class III–V patients. This discriminative trend validates the model's clinical utility. Furthermore, the absolute AKI rates in our high-risk group remain consistent with reports from specific high-risk surgeries [22]. The overall higher incidence compared to the 2009 derivation cohort likely stems from our older patient population, more major surgeries, and more intensive creatinine monitoring. For consistency, AKI was defined using the KDIGO criteria, which offers higher sensitivity and is widely adopted in perioperative studies [23].

The scarcity of effective treatments for AKI highlights the critical need for prophylactic approaches. Our study showed that intraoperative dexmedetomidine reduced the rate of postoperative AKI in elderly, high-risk patients undergoing major non-cardiac surgery. Three recent meta-analyses also found that the perioperative dexmedetomidine lowered AKI incidence in cardiac-surgical patients, especially when the infusion was started immediately after anesthesia induction and continued throughout surgery in elderly patients [11, 12, 24]. However, evidence for dexmedetomidine’s effect on AKI after non-cardiac surgery remains inconsistent. The apparent inconsistencies in reported findings can be attributed to three main factors. Firstly, the timing and dosage of dexmedetomidine administration critically influence drug efficacy. Supporting this, a recent retrospective study demonstrated a significant reduction in postoperative AKI specifically in the subgroup that received a loading dose, whereas no such association was observed in subgroups receiving only a high- or low-dose infusion without a loading dose [25]. Our protocol initiated the loading infusion earlier during the high-surgical-stress phase and maintained it to cover the key pathophysiological window for AKI. This likely explains the positive outcomes in our high-risk cohort. Secondly, patient susceptibility represents another pivotal factor. Most previous studies included non-stratified populations where low-risk patients diluted treatment effects [17]. In contrast, our study prospectively stratified patients according to their AKI risk using a validated index. Our findings—that the benefit was confined to the high-risk group while absent in the low-risk group—directly address these contradictions. Thirdly, previous studies have mainly consisted of retrospective analyses and small-scale randomized controlled trials that included both high- and low-risk patients [17, 26]. Although our present study is a secondary analysis, the parent trial was a prospective, randomized, double-blind, placebo-controlled study with a large sample size. Nevertheless, further large-scale randomized controlled trials are warranted to definitively validate these findings [21].

The renoprotective effects of dexmedetomidine have been extensively investigated in many studies. Firstly, dexmedetomidine can reduce the sympathetic adrenal hyperactivity due to surgical stress [27], and thus improve glomerular filtration and renal blood flow [28]. In IR-induced acute kidney injury rat models, pretreatment with dexmedetomidine improved renal microcirculation [29]. In this study, higher perioperative urine output was found in dexmedetomidine groups in both low-risk and high-risk patients, which was accorded with this mechanism. Secondly, dexmedetomidine can attenuate the systematic inflammatory response by down-regulation of pro-inflammatory cytokines (TNF-α, IL-6, IL-1β) and mitigation of tubular-interstitial inflammation [30]. It modulates key inflammatory signaling pathways, including inhibiting the Janus kinase and signal transducer and activator of transcription (JAK/STAT) signaling pathway [31], down-regulating the activity of Toll like receptor 4 (TLR-4) [32, 33] and nuclear transcription factor B (NF-κB) [34]. Thirdly, dexmedetomidine can alleviate oxidative stress injury by activating the α2-AR/SIRT1/PGC-1α pathway, inhibiting Drp1-mediated mitochondrial fission, and promoting mitochondrial fusion [35]. Fourthly, dexmedetomidine reduced renal tubular apoptosis by reducing activation of p53 and caspase-3 [30]. Basic research has also found that dexmedetomidine exerts anti-apoptotic effects through an α7 nAChR-dependent signaling pathway [36] and through the inhibition of GJ activity by reducing Cx32 protein levels [10]. Fifthly, dexmedetomidine can enhance autophagy through α2-AR-dependent signaling, which upregulates the autophagy inducer AMPK and downregulates the autophagy inhibitor mTOR [9]. In summary, dexmedetomidine mitigates renal injury by suppressing sympathetic overactivity, exerting anti-inflammatory effects, promoting autophagy, reducing oxidative stress, and inhibiting apoptosis.

However, because of the low incidence of severe AKI, the present study observed only a reduction in stage 1 AKI with dexmedetomidine, without a measurable decrease in higher-grade kidney injury. Previous study showed that even stage-1 AKI also prolonged length of stay, increased healthcare costs by $4391 (95% CI $2498 to $6285, *P* < 0.001), and nearly doubled 1-year mortality (OR 1.9, 95% CI 1.3–2.6, *P* < 0.001) [37].Given the high baseline incidence of Stage I AKI, the renoprotective effect of dexmedetomidine still yields tangible short-term benefits and may reduce long-term renal damage and mortality—findings that are clinically meaningful. We also found that higher charlson comorbidity index and urological surgery were independent risk factors of AKI. This result was also supported by other studies [38, 39]. Besides, lower doses of propofol and sufentanil in the dexmedetomidine group reflect its sympatholytic, hemodynamic-stabilizing properties rather than representing a confounder. This reduced anesthetic requirement may thus be a mechanism underlying its renoprotective effect.

Several limitations existed in our study. Firstly, as a secondary analysis, the fixed sample size might be underpowered for the current objective. This implies a risk of overestimating the effect size and limits the ability to perform extensive subgroup analyses. Nevertheless, the identification of a statistically significant effect under these conditions suggests a clinically relevant and robust signal. Therefore, these findings warrant further validation in dedicated prospective trials to draw definitive conclusions. Secondly, Our AKI definition, lacking urine output criteria, limited detection of non-oliguric cases and may have reduced incidence estimates—a limitation common to retrospective designs [25]. Nonetheless, as serum creatinine remains the diagnostic standard, we believe the identified renoprotective signal for dexmedetomidine is fundamentally unchanged. Verification in prospective cohorts with comprehensive AKI criteria is the necessary next step. Thirdly, inflammatory and oxidative biomarkers were not tested in this study. This limited to explore the potential mechanism why dexmedetomidine could decrease the risk of AKI.

## Conclusion

Intraoperative dexmedetomidine was associated with a lower risk of AKI in high-risk but not in low-risk patients undergoing non-cardiac surgery. High-quality trials with larger sample size are needed to validate this hypothesis.

## Supplementary Information

Below is the link to the electronic supplementary material.

Supplementary file1 (DOCX 39 KB)

## Data Availability

The datasets generated and/or analyzed during the current study are not publicly available due to local policies, but are available from the corresponding author on reasonable request.
