# Supplementary material for: Dexmedetomidine reduces acute kidney injury in high-risk but not low-risk patients after non-cardiac surgery: secondary analysis of a randomized controlled trial
Source: J Anesth. 2026 Jan 17;40(5):726–36. doi: 10.1007/s00540-026-03661-8 (PMC13624015; doi:10.1007/s00540-026-03661-8)
Supplement: Supplementary file 1 [file 540_2026_3661_MOESM1_ESM.docx]

**Supplementary Table S1. Demographic and baseline data between low-risk and high-risk patients.**

|  | **Low-risk patients (n=401)** | **High-risk patients (n=210)** | ***P* value** |
| --- | --- | --- | --- |
| Age, years | 67 (63, 74) | 69 (64, 75) | **0.029** |
| Female sex | 207 (51.6) | 36 (17.1) | **<0.001** |
| Body mass index, kg⋅m^-2^ | 23.83 (21.48, 26.16) | 24.80 (22.49, 26.83) | **0.002** |
| Preoperative comorbidity |  |  |  |
| Hypertension | 123 (30.7) | 163 (77.6) | **<0.001** |
| Coronary heart disease | 43 (10.7) | 45 (21.4) | **<0.001** |
| Previous stroke | 32 (8.0) | 28 (13.3) | **0.035** |
| Arrhythmia | 31 (7.7) | 23 (11.0) | 0.183 |
| COPD | 3 (0.7) | 6 (2.9) | 0.089 |
| Diabetes mellitus | 33 (8.2) | 98 (46.7) | **<0.001** |
| Renal dysfunction ^a^ | 4 (1.0) | 11 (5.2) | **0.001** |
| Chronic smoking ^b^ | 94 (23.4) | 60 (28.6) | 0.165 |
| Alcoholism ^c^ | 46 (11.5) | 37 (17.6) | **0.035** |
| Preoperative medication |  |  |  |
| ACEI/ARB | 48 (12.0) | 49 (23.3) | **<0.001** |
| Aspirin | 19 (4.7) | 20 (9.5) | **0.022** |
| NYHA classification |  |  | **0.004** |
| Ⅰ | 296 (73.8) | 131 (62.4) |  |
| Ⅱ | 103 (25.7) | 79 (37.6) |  |
| Ⅲ | 2 (0.5) | 0 (0) |  |
| ASA classification |  |  | **<0.001** |
| Ⅰ | 70 (17.5) | 7 (3.3) |  |
| Ⅱ | 312 (77.8) | 158 (75.2) |  |
| Ⅲ | 19 (4.7) | 45 (21.4) |  |
| CCI^d^ | 4 (4, 5) | 5 (4, 5) | **<0.001** |
| Serum Creatine (ummol/L) | 83.0 (73.0, 91.7) | 96.0 (86.0, 108.0) | **<0.001** |
| eGFR (ml/min/1.73m^2^) | 63.07 (51.93, 75.27) | 53.49 (43.17, 65.83) | **<0.001** |
| Preoperative assessment, point |  |  |  |
| AKI risk class ^e^, class | 2 (1, 2) | 3 (3, 4) | **<0.001** |

Data are median (interquartile range), n (%), or mean ± SD. Numbers in square brackets indicate patients with missing data. P values in bold indicate <0.05.

COPD, Chronic obstructive pulmonary disease; ACEI, angiotensin-converting enzyme inhibitor; ARB, angiotensin receptor blocker; NYHA, New York Heart Association; ASA, American Society of Anesthesiologists.; CCI, Charlson Comorbidity Index; eGFR, estimated Glomerular Filtration Rate.

^a^ Glomerular Filtration Rate lower than 30 ml/min/1.73m^2^.

^b^ Smoking half a pack (10 cigarettes)/day for at least 1 year, either former or current smoker.

^c^ Two drinks or more daily or weekly consumption of the equivalent of 150 ml of alcohol.

^d^ Including age-adjusted.

^e^ Based on General Surgery Acute Kidney Injury Risk Index

**Supplementary Table S2. Intraoperative and postoperative data between low-risk and high-risk patients.**

|  | **Low-risk patients (n=401)** | **High-risk patients (n=210)** | ***P* value** |
| --- | --- | --- | --- |
| **Intraoperative data** |  |  |  |
| Duration of anesthesia, min | 286 (210, 359) | 270 (207, 357) | 0.269 |
| Duration of surgery, min | 204 (138, 274) | 186 (138, 273) | 0.660 |
| Site of surgery |  |  | **<0.001** |
| Intrathoracic | 96 (23.9) | 11 (5.2) |  |
| Intraabdominal | 228 (56.9) | 194 (92.4) |  |
| Spinal and extremital | 77 (19.2) | 5 (2.4) |  |
| Urological surgery ^a^ | 79 (19.7) | 91 (43.3) | **<0.001** |
| Type of surgery |  |  | **<0.001** |
| Open | 111 (27.7) | 23 (11.0) |  |
| Mini-invasive ^b^ | 290 (72.3) | 187 (89.0) |  |
| Medications during anesthesia |  |  |  |
| Dosage of study drug, μg | 118 (90, 151) | 120 (95, 157) | 0.393 |
| Dose of propofol, mg | 874 (612, 1200) | 864 (665, 1200) | 0.365 |
| Dose of sufentanil, μg | 76 (57, 99) | 73 (56, 101) | 0.904 |
| Use of vasopressors ^c^ | 147 (36.7) | 60 (28.6) | **0.045** |
| Use of vasodilators ^d^ | 50 (12.5) | 27 (12.9) | 0.891 |
| Use of NSAIDs ^e^ | 14 (3.5) | 7 (3.3) | 0.919 |
| Use of 5-HT3 receptor antagonists ^f^ | 350 (87.3) | 183 (87.1) | 0.961 |
| Use of low-dose glucocorticoids ^g^ | 387 (96.5) | 204 (97.1) | 0.676 |
| Total infusion, ml | 2250 (1600, 3100) | 2513 (1700, 3413) | 0.106 |
| Use of colloids, ml ^h^ | 0 (0, 500) | 0 (0, 500) | 0.209 |
| Blood transfusion | 75 (18.7) | 25 (11.9) | **0.031** |
| Estimated blood loss, ml | 100 (50, 300) | 100 (50, 300) | 0.097 |
| Urine output, ml | 500 (300, 800) | 400 (200, 700) | **0.003** |
| Mean BIS value (%) ^i^ | 54 (50, 58) | 53 (49, 57) | 0.074 |
| Adverse events |  |  |  |
| Hypotension | 165 (41.1) | 73 (34.8) | 0.124 |
| Hypertension | 58 (14.5) | 28 (13.3) | 0.703 |
| Arrhythmia | 7 (1.7) | 5 (2.4) | 0.818 |
| **Postoperative data** |  |  |  |
| PCIA morphine, mg | 49 (40, 50) | 50 (42, 50) | 0.102 |
| Supplemental analgesics within 5 days |  |  |  |
| Use of NSAIDs ^j^ | 200 (49.9) | 63 (30.0) | **<0.001** |
| Use of oral tramadol | 8 (2.0) | 4 (1.9) | >0.999 |
| Other sedatives within 5 days |  |  |  |
| Use of midazolam | 13 (3.2) | 6 (2.9) | 0.795 |
| Use of dexmedetomidine | 7 (1.7) | 9 (4.3) | 0.062 |
| Use of propofol | 1 (0.2) | 0 (0) | >0.999 |

Data are median (interquartile range), n (%) or mean ± SD. Numbers in square brackets indicate patients with missing data. P value in bold indicates <0.05.

NSAIDs, non-steroid anti-inflammatory drugs; 5-HT3, 5-hydroxytryptamine type 3; BIS, Bispectral Index; PCIA, patient-controlled intravenous analgesia;

^a^ Included radical nephrectomy, partial nephrectomy, radical nephroureterectomy, radical cystectomy, ureteral reimplantation, radical prostatectomy.

^b^ Included laparoscopic and thoracoscopic surgery.

^c^ Included ephedrine, phenylephrine, metaraminol, norepinephrine, epinephrine and dopamine.

^d^ Included nicardipine and urapidil.

^e^ Included flurbiprofen axetil (50-100 mg) and parecoxib (40 mg).

^f^ Included tropisetron (5 mg) and ondansetron (4 mg).

^g^ Dexamethasone (5–10 mg) or methylprednisolone (40 mg) for the prevention of postoperative nausea and vomiting.

^h^ Included hydroxyethyl starch and/or succinylated gelatin.

^i^ Monitored by Bispectral Index (BIS) with data collected at 1-min intervals from end of anaesthesia induction to end of surgery.

^j^ Included parecoxib (40 mg) or flurbiprofen axetil (50 mg), administered within 5 days of surgery.

**Supplementary Table S3 Individual non-AKI complications within 30 days of surgery**

|  | **Low-risk patients (n=401)** | | | | **High-risk patients (n=210)** | | | |
| --- | --- | --- | --- | --- | --- | --- | --- | --- |
|  | **Dexmedetomidine (n=203)** | **Placebo**  **(n=198)** | **Median difference, RR, or HR (95% CI) ^a^** | ***P* value** | **Dexmedetomidine (n=103)** | **Placebo**  **(n=107)** | **Median difference, RR, or HR (95% CI) ^a^** | ***P* value** |
| Central nervous system, n (%) | 2 (1.0) | 0 (0) | - | 0.499 | 1 (1.0) | 1 (0.9) | 1.04 (0.07, 16.39) | >0.999 |
| Ischemic stroke ^a^ | 0 (0) | 0 (0) |  | >0.999 | 1 (1.0) | 0 (0) |  | 0.490 |
| Transient ischemic accident ^b^ | 2 (1.0) | 0 (0) |  | 0.499 | 0 (0) | 1 (0.9) |  | >0.999 |
| Cardiovascular system, n (%) | 17(8.4) | 19(9.6) | 0.87 (0.47, 1.63) | 0.669 | 5(4.9) | 9(8.4) | 0.58 (0.20, 1.66) | 0.302 |
| Acute coronary syndrome ^c^ | 7 (3.4) | 10 (5.1) |  | 0.426 | 3 (2.9) | 7 (6.5) |  | 0.363 |
| Congestive heart failure ^d^ | 3 (1.5) | 1 (0.5) |  | 0.633 | 2 (1.9) | 0 (0) |  | 0.239 |
| New onset arrhythmia ^e^ | 5(2.5) | 9(4.5) |  | 0.256 | 2(1.9) | 2(1.9) |  | >0.999 |
| Deep venous thrombosis ^f^ | 0 (0) | 4 (2.0) | - | 0.059 | 1 (1.0) | 1 (0.9) | 1.04 (0.07, 16.39) | >0.999 |
| Respiratory system, n (%) | 2 (1.0) | 2 (1.0) | 0.98 (0.14, 6.86) | 0.980 | 0 (0) | 1 (0.9) | - | >0.999 |
| Pneumonia ^g^ | 1 (0.5) | 1 (0.5) |  | >0.999 | 0 (0) | 0 (0) |  | >0.999 |
| Respiratory failure ^h^ | 1 (0.5) | 0 (0) |  | >0.999 | 0 (0) | 1 (0.9) |  | >0.999 |
| Asthma ^i^ | 0 (0) | 1 (0.5) |  | 0.494 | 0 (0) | 0 (0) |  | >0.999 |
| Surgery-related complications, n (%) | 8(3.9) | 10(5.1) | 0.78 (0.31, 1.94) | 0.592 | 4 (3.9) | 8 (7.5) | 0.52 (0.16, 1.67) | 0.262 |
| Gastrointestinal hemorrhage ^j^ | 0 (0) | 2 (1.0) |  | 0.243 | 0 (0) | 4 (3.7) |  | 0.122 |
| Ileus ^k^ | 2 (1.0) | 1 (0.5) |  | >0.999 | 2 (1.9) | 0 (0) |  | 0.239 |
| Anastomotic leak ^l^ | 1 (0.5) | 1 (0.5) |  | >0.999 | 0 (0) | 1 (0.9) |  | >0.999 |
| Surgical site infection ^m^ | 5(2.5) | 1(0.5) |  | >0.999 | 2 (1.9) | 1 (0.9) |  | 0.973 |
| Sepsis ^n^ | 0 (0) | 6 (3.0) |  | 0.014 | 0 (0) | 2 (1.9) |  | 0.498 |

Data are n (%). Numbers in square brackets indicate patients with missing data. P value in bold indicates <0.05. RR= Relative Risk; CI= Confidence Interval, HR= Hazard Ratio; AKI, Acute kidney injury.

^a^ Persisted (more than 24 hours) new focal neurologic deficit and confirmed by neurologic imaging.

^b^ Episodes of neurological dysfunction caused by focal cerebral ischemia and relieved within 24 hours. Diagnoses were confirmed by neurologists.

^c^ Included myocardial infarction and unstable angina, which was confirmed by clinical symptoms, electrocardiogram and blood tests of cardiac markers.

^d^ Defined as the presence of symptoms (i.e. shortness of breathing) and signs (i.e. chest X-rays findings) suggesting heart failure, a serum BNP > 400 pg/ml, and a typical clinical response to diuretic therapy.

^e^ Included sinus bradycardia, sinus tachycardia, atrial fibrillation and ventricular arrhythmia, which is diagnosed by electrocardiographic monitoring and required therapeutic intervention.

^f^ Confirmed by lower limbs venous ultrasonography and required anticoagulant therapy.

^g^ Newly infiltrate on chest x-ray combined with hyperthermia (>38℃) and leukocytosis and required intravenous antibiotic therapy.

^h^ Requirement of mechanical ventilation of more than 24 hours.

^i^ Diagnosed according to clinical signs and required inhaled bronchodilator therapy.

^j^ Decrease of hemoglobin level combined with positive gastrointestinal occult blood test results that required treatment.

^k^ Lack of bowel movement, flatulence, and requirement of intravenous fluid therapy for more than one week after surgery.

^l^ Extravasation of intestinal content or contrast agent in the body cavity or retroperitoneal space that required percutaneous drainage.

^m^ Included superficial incisional and deep incisional surgical site infection, which is confirmed by bacteria culture.

^n^ Two or more criteria of systemic inflammatory response syndrome, with suspected or known infection.
